# Effects of canola or olive oil on plasma lipids, lipoprotein-associated phospholipase A_2_ and inflammatory cytokines in patients referred for coronary angiography

**DOI:** 10.1186/s12944-020-01362-z

**Published:** 2020-08-14

**Authors:** Nafiseh Khandouzi, Ali Zahedmehr, Javad Nasrollahzadeh

**Affiliations:** 1 Department of Clinical Nutrition & Dietetics, National Nutrition, and Food Technology Research Institute, Faculty of Nutrition Sciences and Food Technology, Shahid Beheshti University of Medical Sciences, No. 7, Hafezi St., Farahzadi Blvd., Qods Town, Tehran, Iran; 2 Cardiovascular Intervention Research Center, Rajaie Cardiovascular, Medical & Research Center, Iran University of Medical Sciences, Tehran, Iran

**Keywords:** Olive oil, Canola oil, Lipid profile, Lipoprotein-associated phospholipase A_2_, Inflammatory markers

## Abstract

**Background:**

The potential cardioprotective benefits of olive oil (OO) and canola oil (CO) consumption have been shown in some studies. The present study compared the effects of CO and OO on plasma lipids, some inflammatory cytokines, and lipoprotein-associated phospholipase A_2_ (Lp-PLA_2_) mass and activity in patients undergoing coronary angiography.

**Methods:**

The current randomized, controlled, parallel-arm, clinical trial involved 48 patients (44 men and 4 women, aged 57.63 ± 6.34 years) with at least one classic cardiovascular risk factor (hypertension, dyslipidemia, or diabetes) who referred for coronary angiography. Patients were randomly divided into two groups and received 25 mL/day refined olive oil (*n* = 24) or canola oil (*n* = 24) for 6 weeks. Plasma lipids, some selected inflammatory markers, and Lp-PLA_2_ levels were measured at baseline and after the intervention.

**Results:**

CO consumption produced a significant reduction in plasma Lp-PLA_2_ mass (− 0.97 ± 1.84 vs. 0.34 ± 1.57 ng/mL, *p* = 0.008 for CO and OO, respectively), whereas the mean changes in interleukine-6 concentration were significantly lower after OO consumption compared with CO (− 9.46 ± 9.46 vs. -0.90 ± 6.80 pg/mL, *p* = 0.008 for OO and CO, respectively). After 6 weeks of intervention, no significant changes were observed in plasma Lp-PLA_2_ activity, complement C3, C4, or lipid profiles in the two intervention groups.

**Conclusions:**

Comparing the two vegetable oils in subjects with cardiovascular risk factors showed that the consumption of olive oil is more effective in reducing the level of inflammatory cytokine interleukine-6, whereas canola oil was more effective in lowering Lp-PLA_2_ levels; however, this finding should be interpreted with caution, because Lp-PLA_2_ activity did not change significantly.

**Trial registration:**

IRCT20160702028742N5 at www.irct.ir (04/19/2019).

## Background

Lifestyle modification, including a healthy diet, is the first therapeutic step to reducing cardiovascular disease (CVD) [1]. Reducing the amount of dietary saturated fatty acids (SFAs) and replacing them with unsaturated fats are among the main nutritional recommendations for the prevention and treatment of CVD [2]. Inclusion of vegetable oils rich in monounsaturated fatty acids (MUFAs) in the diet has been associated with several cardioprotective effects [3]. Olive and canola oils, two commonly consumed vegetable oils, are low in SFAs but rich in MUFAs, and both are recommended to be included in a healthy diet for cardioprotection [4, 5].

The potential cardioprotective benefits of olive oil (OO) consumption, especially in the Mediterranean diet, have been extensively studied [4, 6]. Scientific evidence suggests, however, that replacing SFAs with polyunsaturated fatty acids (PUFAs) may reduce CVD risk factors somewhat more than replacing SFAs with MUFAs [2]. In addition to their beneficial effects, PUFAs have been shown to be safe [7]. Therefore, oils with low SFAs that contain relatively high levels of PUFAs as well as MUFAs may be preferable for improving cardiovascular risk factors. Canola oil (CO) which contains low amounts of SFAs, high amounts of MUFAs, and relatively high amounts of PUFAs could be a reasonable choice for inclusion in a healthy diet to replace SFAs and increase unsaturated fats intake [8]. Evidence supports several potential health benefits of canola oil consumption in terms of reducing cardiovascular risk factors and improving health [5]. The comparison of canola and olive oils showed that the MUFA content of OO is slightly higher, while the amount of PUFAs is higher in CO [5].

In addition to the classic CVD risk factors such as plasma lipid and lipoprotein concentrations, measuring inflammatory biomarkers is useful for cardiovascular risk assessment and predicting cardiovascular risk [9, 10]. Numerous inflammatory biomarkers are implicated in atherosclerosis; each one increases our understanding of this complex process. Interleukin-6 (IL-6) is among the well-studied inflammatory biomarkers related to cardiovascular risk [10–13]. Lipoprotein-associated phospholipase A_2_ (Lp-PLA_2_) is a member of the phospholipase A_2_ family produced by inflammatory cells and mediates vascular inflammation [14]. Elevated plasma Lp-PLA_2_ activity is positively correlated with an increase in inflammatory cytokines, particularly IL-6 [15]. A meta-analysis of the prospective studies of Lp-PLA_2_ showed an association between Lp-PLA_2_ activity and mass and a worse prognosis of coronary artery disease (CAD), ischemic stroke, and vascular mortality [16]. In particular, a higher level of Lp-PLA_2_ activity may imply a worse cardiovascular prognosis in high-risk patients referred for coronary angiography [17]. In addition to the effects that fatty acid intake can have on the concentrations of lipids and inflammatory markers, they may affect the level of plasma C3 [14], which, itself, has been associated with atherosclerosis and cardiovascular risk factors [18].

Research on the impacts of olive and canola oils on cardiovascular biomarkers, particularly on inflammatory biomarkers, is scarce. Therefore, the present study aimed to examine the effects of CO and OO on plasma lipids, inflammatory cytokines, Lp-PLA_2_ mass and activity, and complement C3 and C4 concentrations in individuals undergoing coronary angiography.

## Materials and methods

### Participants

The participants were patients who referred for coronary angiography to the Shahid Rajaei Cardiovascular, Medical & Research Center, Tehran, Iran. Patients entered the study 1 month after undergoing angiography to ensure that their medical and pharmacological status was stable.

Eligible subjects were men and post-menopausal women less than 75 years of age who had at least one major cardiovascular risk factor, such as hypertension, diabetes mellitus, dyslipidemia, or acute cardiac event. The exclusion criteria were the regular use of anti-inflammatory medication, dietary antioxidants, or omega-3 supplements during the month prior to the study; any changes in the disease treatment plan, including type or dose of drugs or coronary artery bypass graft (CABG); and gastrointestinal complications such as diarrhea during the study. In addition, participants with low adherence to the intervention (who consumed less than 80% of the olive or canola oils delivered at baseline) were excluded from the study.

### Study design

The present study was a randomized, controlled, open-label, parallel-arm clinical trial conducted in the spring and summer of 2019 in Tehran, Iran. The enrollment period ran from June 18, 2019 to September 15, 2019. At baseline, demographic and medical information was obtained through face-to-face interviews and reviews of medical files, respectively. Cardiovascular risk factors (hypertension, diabetes, and dyslipidemia) and past medical histories were determined from the patients’ medical records. All participants received dietary advice on a heart-healthy diet at baseline. Daily consumption of at least five servings of fruits and vegetables, substituting lower-fat dairy products and meats for higher fats ones, and lower use of salt and simple sugars were among the dietary advice provided. Next, the participants were randomly assigned to one of the two groups in a 1:1 ratio following simple randomization procedures using computerized random numbers by one of the study researchers not involved in patient care. Participants were requested to consume a daily amount of 25 mL of refined olive oil (OO) (Etka, Roodbar, Iran) or canola oil (CO) (CanaPlus, British Columbia, Canada) raw with meals for 6 weeks. The fatty acid composition of OO and CO is shown in Table 1. Olive and canola oils were provided to patients in sufficient quantities. To ensure compliance with the intervention and proper oil consumption, participants were followed up weekly by telephone contact. If a participant consumed the recommended amount of oil (25 ml per day) less than 5 days a week, s/he was excluded from the study.

**Table 1:** Chemical composition of oils

Chemical Component	Canola Oil	Refined Olive Oil
**Fatty Acid (per 100 g):**		
**Total SFAs**	6.40	17.87
**Total MUFA**	61.37	66.68
Oleic acid	59.36	65.70
**Total PUFAs**	29.20	11.75
Linoleic acid	20.26	11.23
Alpha-linolenic acid	8.48	0.48

The procedures followed in this trial were in accordance with the 1964 Helsinki Declaration, and the study protocol was approved by the Ethics Committee of the National Nutrition & Food Technology Research Institute, Tehran, Iran (No. IR.SBMU.NNFTRI.REC.1398.074). Written informed consent was obtained from all participants prior to beginning the study. This clinical trial was registered at the Iranian Registry Center of Clinical Trials (IRCT) (registration number: 20160702028742 N5).

### Anthropometric measures

Participant weight and height were measured at baseline and after 6 weeks of intervention by the study dietitian. Weight was measured without shoes, coats, or jackets using a digital scale. Height was measured without shoes using a wall-mounted stadiometer.

### Biochemical parameters

Venous blood samples were obtained from each patient after 12-h overnight fasting and collected into heparinized tubes at baseline and after 6 weeks of intervention. The blood samples were centrifuged (4000 rpm for 20 min), and the resulting plasma was stored at − 80 °C. Plasma Lp-PLA_2_ mass and activity were analyzed by a commercially available ELISA kit (ZellBio GmbH, Ulm, Germany) and commercial colorimetric assay kit (Cayman Chemical, Ann Arbor, MI, USA), respectively. A commercially available ELISA kit (Biolegend, San Diego, CA, USA) was used to measure plasma IL-6 concentration. Plasma lipids and lipoproteins were analyzed using the colorimetry method with an auto-analyzer (Selectra 2, Vital Scientific, Spankeren, The Netherlands) using commercial kits (Pars Azmoon, Karaj, Iran). Low-density lipoprotein-cholesterol (LDL-C) was measured using a direct enzymatic method. The small-dense LDL-cholesterol content in plasma was quantitated according to the method described previously [19]. Non-high density lipoprotein-cholesterol (Non-HDL-C) was calculated by subtracting HDL-C from total cholesterol. Plasma levels of complement C3 and C4 were determined using the *turbidimetric* method with commercial kits (Pars Azmoon, Karaj, Iran) using an auto-analyzer.

### Dietary intakes and physical activity

Dietary intake and physical activity levels were monitored at baseline and after 6 weeks. Dietary intake was assessed using the 24-h dietary recall questionnaire completed in 3 days (two regular days in the middle of the week and 1 day on the weekend) by a trained dietitian. Participants were asked to maintain their habitual lifestyle throughout the study. Recall data was analyzed using the Nutritionist software (version IV, N-Squared Computing, San Bruno, CA, USA) to which was added the local food data.

### Statistical analysis

Although the primary outcome was Lp-PLA_2_, sample size could not be calculated based on this variable, because according to our search, no study has compared the effects of CO and OO on Lp-PLA_2_ mass or activity. Nonetheless, studies have shown that Lp-PLA_2_ transported in plasma is predominantly (80%) associated with LDL-C [20]. Therefore, the study sample size was calculated using LDL-C as the primary outcome variable. To detect a change in the mean of LDL-C concentration (10 mg/dL) as reported in a previous investigation [21] at the 5% level of significance and with 80% power, 24 participants were needed in each arm of the two-arm trial.

Data was analyzed using SPSS software for Windows version 21 (SPSS Inc., Chicago, IL, USA). All values were reported as mean ± SD or percentage (%). The per-protocol analysis was performed (i.e., only those who completed the study were included in the analyses). The normality of distribution of the study variables was tested by the Shapiro-Wilk test. When the variables were not normally distributed, raw values were log-transformed. Analysis of covariance (ANCOVA) was used to compare the 6-week values between the groups using the baseline measures as the covariate. Paired samples *t*-test was used to compare the measurements in the beginning and at the end of the intervention within the study groups. The χ^2^ test was used to compare categorical variables. The statistical significance level was set at *p* = 0.05 (two tails).

## Results

**Fig. 1:**
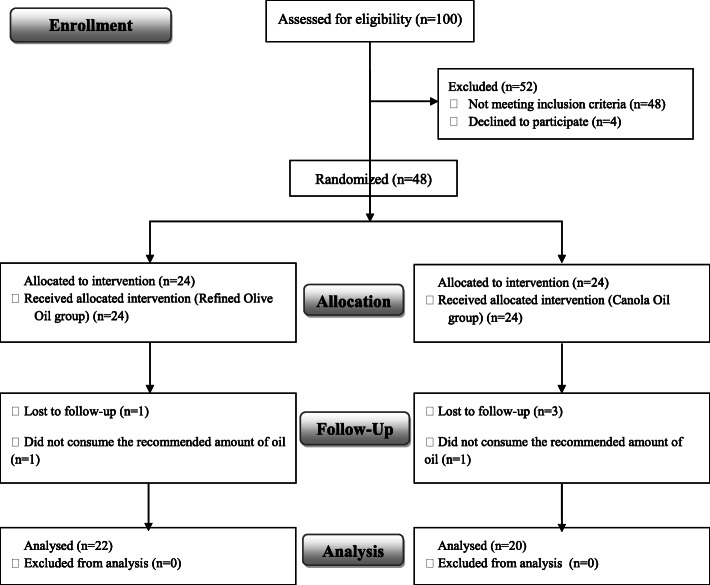
Consort flow diagram of selection and allocation of the participants included in the study

A total of 100 patients were screened for eligibility, and 48 patients entered the study. Six patients were excluded during the intervention due to low adherence to dietary intervention, travel, and refusal to continue. Therefore, the final study population comprised 42 subjects: 22 subjects in the OO group, and 20 subjects in the CO group (Fig. 1). Table 2 summarizes the general characteristics of the participants. No significant differences were observed in baseline characteristics between the two groups.

**Table 2:** Baseline characteristics of the participants ^a^

Variable	Olive Oil group (***n*** = 22)	Canola Oil group (***n*** = 20)	***P***-value ^**b**^
**Age (year)**	55.09 ± 6.92	59.30 ± 5.84	0.44
**Sex (Male)**	20 (91%)	18 (90%)	0.66
**PCI history**	16 (73%)	16 (80%)	0.89
**CABG history**	2 (9.1%)	1 (5%)	0.86
**Diabetes Mellitus**	8 (36%)	8 (40%)	0.91
**Hypertension**	11 (50%)	11 (55%)	0.92
**Dyslipidemia**	12 (54%)	8 (40%)	0.55
**Overweight**	16 (73%)	11 (55%)	0.19
**Smoking**	8 (36%)	4 (20%)	0.30
**Medications:**			
**Aspirin**	21 (95%)	20 (100%)	0.93
**Clopidogrel**	15 (68%)	10 (50%)	0.29
**Statins**	20 (91%)	19 (95%)	0.93
**ACEI**	9 (41%)	4 (20%)	0.12
**ARB**	10 (45%)	13 (65%)	0.42
**BB**	15 (68%)	15 (75%)	0.81
**Nitrates**	7 (31%)	7 (35%)	0.90
**Aldosteron Antagonist**	5 (22%)	1 (5%)	0.17

^a^Data presentedas Mean ± SD or number (percentage)

^b^Data were compared using Independent t-testor Chi-square test

*PCI* Percutaneous Coronary Intervention, *CABG* Coronary Artery Bypass Graft, *ACEI* Angiotensin Converting Enzyme Inhibitor, *ARB* Angiotensin Receptor Blocker, *BB* Beta-Blocker

Baseline levels and 6-week changes in anthropometric measures are shown in Table 3. There were no significant differences between the OO and CO groups concerning body weight or physical activity at baseline and after intervention.

**Table 3:** Anthropometric measures and physical activity of the participants^a^

Variable	Time	Olive Oil group (***n*** = 22)	Canola Oil group (***n*** = 20)	***P***-value^**b**^
**Body Weight (kg)**	Baseline	80.45 ± 11.77	78.65 ± 12.81	0.62
	6-week	80.45 ± 11.49	78.30 ± 12.47	0.64
**BMI (kg/m**^**2**^**)**	Baseline	26.91 ± 3.38	27.31 ± 4.39	0.34
	6-week	26.94 ± 3.35	27.19 ± 4.31	0.30
**Physical Activity (MET-h/day)**	Baseline	27.99 ± 5.23	27.30 ± 3.49	0.65
	6-week	28.01 ± 5.23	27.36 ± 3.57	0.69

^a^All values are Mean ± SD

^b^Data were compared using Independent t-test

*BMI* Body Mass Index, *MET* Metabolic Equivalent

As shown in Table 4, energy and dietary intakes of nutrients did not differ between the groups at baseline. Dietary intakes of total fat and monounsaturated fat in both groups increased during the intervention due to the consumption of olive and canola oils. However, there were no differences in the dietary intakes of the two groups at 6 weeks after intervention.

**Table 4:** Dietary intake of the participants at the baseline and after the intervention ^a^

Variable	Time	Olive Oil group^**b**^ (***n*** = 22)	Canola Oilgroup^**b**^ (***n*** = 20)	***P***-value ^**c**^
**Energy (kcal)**	Baseline	1781.40 ± 196.10	1767.20 ± 146.19	0.55
	6-week	1850.72 ± 143.20	1801.20 ± 140.44	0.26
**Carbohydrate (g)**	Baseline	233.05 ± 32.18	235.20 ± 28.75	0.82
	6-week	234.25 ± 33.57	232.85 ± 29.33	0.73
**Carbohydrate (%)**	Baseline	51.90 ± 4.60	53.26 ± 5.16	0.38
	6-week	50.47 ± 4.20	51.24 ± 4.97	0.60
**Protein (g)**	Baseline	69.75 ± 15.55	74.25 ± 15.12	0.36
	6-week	70.65 ± 11.27	73.30 ± 11.47	0.46
**Protein (%)**	Baseline	15.51 ± 3.05	16.76 ± 2.84	0.18
	6-week	15.29 ± 2.27	16.26 ± 2.05	0.16
**Total Fat (g)**	Baseline	64.30 ± 11.59	59.05 ± 9.93	0.13
	6-week	71.40 ± 7.91 ^†^	68.90 ± 8.36^†^	0.33
**Total Fat (%)**	Baseline	32.17 ± 4.53	30.05 ± 4.15	0.13
	6-week	34.87 ± 4.36 ^†^	34.47 ± 3.83^†^	0.75
**SFA (g)**	Baseline	16.00 ± 4.56	16.10 ± 5.09	0.94
	6-week	14.90 ± 3.56	13.75 ± 3.37	0.30
**SFA (%)**	Baseline	7.99 ± 2.01	8.15 ± 2.24	0.82
	6-week	7.28 ± 1.85	6.84 ± 1.44	0.40
**MUFA (g)**	Baseline	19.45 ± 6.73	18.45 ± 4.09	0.26
	6-week	30.30 ± 4.37 ^†^	28.50 ± 3.53^†^	0.18
**MUFA (%)**	Baseline	11.18 ± 2.99	10.38 ± 1.90	0.12
	6-week	14.79 ± 2.45 ^†^	14.25 ± 1.59^†^	0.10
**PUFA (g)**	Baseline	25.10 ± 6.48	22.45 ± 4.11	0.13
	6-week	23.75 ± 4.65	24.40 ± 2.76	0.59
**PUFA (%)**	Baseline	12.58 ± 3.03	11.47 ± 2.21	0.19
	6-week	11.56 ± 2.16	12.22 ± 1.37	0.25
**Omega-3 (mg)**	Baseline	213.30 ± 178.73	167.25 ± 92.56	0.31
	6-week	233.85 ± 150.65	221.55 ± 66.09^†^	0.27
**Cholesterol (mg)**	Baseline	167.65 ± 82.15	170.00 ± 55.65	0.91
	6-week	157.00 ± 73.63	167.35 ± 60.39	0.63
**Fiber (g)**	Baseline	15.45 ± 2.32	14.60 ± 3.78	0.88
	6-week	15.90 ± 2.78	15.75 ± 3.24	0.87

^a^All values are Mean ± SD

^b^ The daily olive or canola oil consumption is considered

^c^Data were compared using Independent t-test

^†^Significantly different from Baseline. Data were compared using Paired t-test

*SFA* Saturated Fatty Acid, *MUFA* Monounsaturated Fatty Acids, *PUFA* Polyunsaturated Fatty Acids

Plasma levels of lipids, lipoproteins, and inflammatory markers are shown in Table 5. The baseline values were not different between the two groups. CO consumption resulted in a significant reduction in plasma Lp-PLA_2_ mass (*p* = 0.008, power = 0.78) during the 6 weeks, whereas the mean changes of IL-6 concentration were significantly lower after OO consumption compared with CO consumption (*p* = 0.008, power = 0.80). After 6 weeks of intervention, plasma Lp-PLA_2_ activity, C3, C4, and lipid profiles had no significant changes in either group (Table 5). Owing to some loss to follow-up, the statistical power to detect differences in LDL-C was decreased (the actual power of test analysis was 0.70).

**Table 5:** Measures of biochemical variables by intervention groups ^a^

Variable	Olive Oil group (***n*** = 22)			Canola Oil group (***n*** = 20)			P ^**c**^
	Baseline	6-Week	Δ value ^**b**^	Baseline	6-Week	Δ value ^**b**^	
**TC (mg/dL)**	116.77 ± 25.15	124.36 ± 28.94	8.45 ± 17.67	136.25 ± 46.45	133.35 ± 47.54	−2.90 ± 16.81	0.10
**LDL-C (mg/dL)**	63.64 ± 13.72	67.36 ± 17.39	3.68 ± 11.27	74.65 ± 30.46	72.90 ± 28.98	−1.75 ± 9.03	0.16
**sd-LDL (mg/dL)**	24.14 ± 10.32	23.14 ± 11.18	−0.91 ± 6.34	22.10 ± 7.51	21.00 ± 9.48	− 1.10 ± 6.35	0.62
**HDL-C (mg/dL)**	37.73 ± 7.92	39.50 ± 7.46	1.50 ± 5.80	42.90 ± 6.49	43.15 ± 8.33	0.35 ± 5.21	0.79
**TC/HDL-C**	3.17 ± 0.47	3.25 ± 0.71	0.08 ± 0.39	3.06 ± 0.72	3.10 ± 0.83	0.03 ± 0.33	0.81
**TG (mg/dL)**	112.73 ± 33.04	120.50 ± 61.38	7.63 ± 36.45	122.45 ± 31.94	126.05 ± 35.40	3.90 ± 20.92	0.32
**Lp-PLA**_**2**_ **mass** **(ng/mL)**	5.66 ± 4.09	6.00 ± 4.51	0.34 ± 1.57	3.96 ± 2.45	2.99 ± 1.65	− 0.97 ± 1.84 ^†^	0.008
**Lp-PLA**_**2**_ **Activity (nmol/min/mL)**	0.022 ± 0.013	0.021 ± 0.017	− 0.000 ± 0.008	0.012 ± 0.003	0.010 ± 0.004	− 0.001 ± 0.002	0.74
**IL-6 (pg/mL)**	21.95 ± 20.38	12.49 ± 13.70	− 9.46 ± 9.46 ^†^	19.53 ± 10.52	18.62 ± 8.55	−0.90 ± 6.80	0.008
**C3 (g/L)**	199.32 ± 35.04	200.68 ± 33.63	1.36 ± 36.64	205.30 ± 44.46	223.50 ± 42.40	18.10 ± 49.00	0.08
**C4 (g/L)**	38.82 ± 11.99	36.23 ± 9.48	−2.59 ± 9.09	39.20 ± 9.03	34.60 ± 10.35	−4.55 ± 12.43	0.52
**C3/C4**	5.56 ± 1.98	5.37 ± 2.08	0.23 ± 0.91	5.37 ± 2.08	6.36 ± 2.45	0.99 ± 2.53	0.27

^a^ All values are Mean ± SD

^b^ Change of parameter between 6-week and baseline (6-week minus baseline)

^c^ The values for 6-week were analyzed using ANCOVA with baseline values as covariate

^†^ Significantly different from baseline. Data were compared using Paired t-test

*TC* Total Cholesterol, *LDL-C* Low Density Lipoprotein-Cholesterol, *sd-LDL* Small Dense LDL, *HDL* High Density Lipoprotein-Cholesterol, *TG* Triglyceride, *Lp-PLA*_*2*_ Lipoprotein-Associated Phospholipase A_2_, *IL-6* Interleukin-6, *C3* Complement component 3, *C4* Complement component 4

## Discussion

**Fig. 2:**
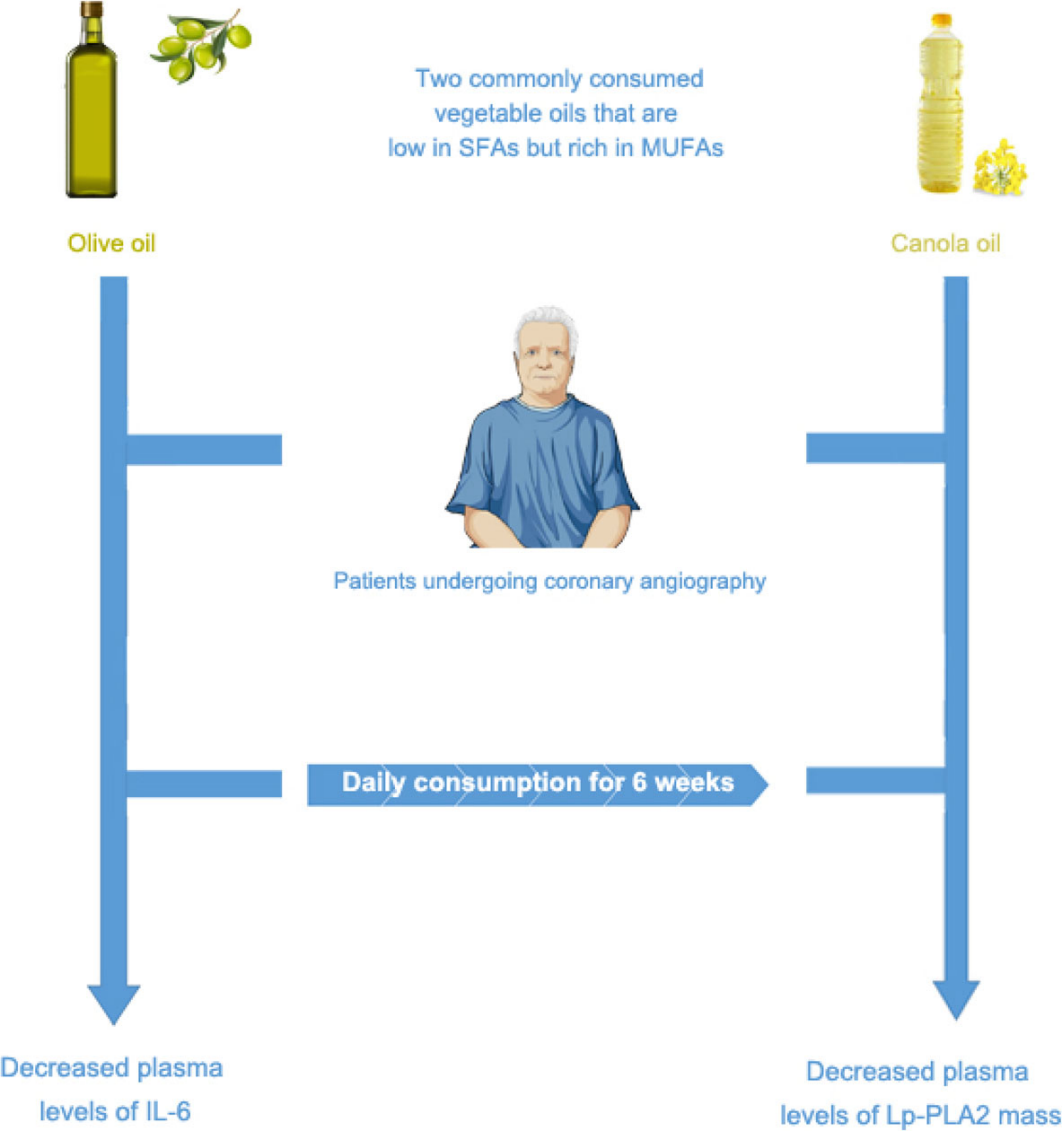
Summary of research rationale and main findings

The present study suggests that a relatively short dietary intervention with refined olive oil can have a significant effect on plasma IL-6 concentration. In contrast, canola oil may impact plasma Lp-PLA_2_ mass without changing lipoproteins or other inflammatory biomarkers in patients referring for coronary angiography (Fig. 2).

In the present study, the consumption of olive oil had a greater lowering effect on plasma IL-6 concentration. The difference in plasma IL-6 levels between the two groups can be related to the difference in fatty acid composition of canola and olive oils. In particular, olive oil consists of 14.5% SFA, 70% oleic acid, 11% linoleic acid, and 1.5% palmitoleic acid, whereas canola oil is characterized by a low level of SFAs (7%); 61% oleic acid, 19% linoleic acid, and 9% alpha-linolenic acid (ALA) [22]. Compared to canola oil, the amount of oleic and palmitoleic acid in olive oil is higher. In vitro studies have shown that the addition of oleic or palmitoleic acid to the cell culture medium is effective in reducing or preventing increases in IL-6 levels [23–26]. Some human studies have shown evidence of an inverse association between oleic acid and IL-6 concentration [27]. In addition, a diet rich in MUFA may also lower thrombotic factors, including the von Willebrand factor [28], the plasma levels of which are increased in high risk populations [29], and its release may be induced by IL-6 [30].

A growing body of evidence indicates that Lp-PLA_2_ represents an independent risk factor for CVD. Lp-PLA_2_ belongs to the family of structurally diverse phospholipase A_2_ enzymes also known as platelet-activating factor acetylhydrolase (PAF-AH) [31]. Circulating Lp-PLA_2_ is primarily associated with LDL-C; the majority of it is bound to atherogenic small-dense LDL-C particles [32]. In the present study, the consumption of CO-reduced plasma Lp-PLA_2_ mass but not of its activity. Previous studies have shown a modest correlation between Lp-PLA_2_ mass and activity (30, 31). It has been suggested that Lp-PLA_2_ is associated with LDL-C in plasma, and this may result in the under-detection of Lp-PLA_2_ mass by the ELISA method (32). However, all study samples were measured in the same way in the current study, thus possible under-detection of the Lp-PLA_2_ mass was the same for the baseline and the final samples. Despite this, both Lp-PLA_2_ mass and activity are predictors of CVD events (33, 34). It has been shown that tocopherols could inhibit Lp-PLA_2_ activity [33]. The amount of alpha-tocopherol found in olive and canola oils is similar; however, canola oil is richer in gamma-tocopherol [34], which may influence the observed effect on Lp-PLA_2_. The observed decrease in Lp-PLA_2_ levels following CO consumption may also be related to its n-3 fatty acid content. Canola oil contains more PUFAs (both n-6 and n-3) and less MUFA than olive oil. In patients admitted to elective coronary angiography, the content of long-chain n-3 PUFA eicosapentaenoic acid (EPA) in adipose tissue was inversely associated with plasma Lp-PLA_2_ mass [35]. Similarly, in participants of the Multi-Ethnic Study of Atherosclerosis, plasma Lp-PLA_2_ mass and activity were significantly lower in those with higher plasma EPA and docosahexaenoic acid (DHA) levels [36]. The evidence from intervention trials examining the influence of n-3 PUFAs on Lp-PLA_2_ is ambiguous. In healthy people, two studies have reported no effect of n-3 PUFA supplementation on Lp-PLA_2_ [37, 38]. Nelson et al. showed that supplementation with n-3 fatty acid capsules (fish oil or flaxseed oil) over 8 weeks had no significant effect on plasma Lp-PLA_2_ mass or activity when compared to the control (olive oil capsule supplementation) [37]. In contrast, Asztalos et al. showed that compared to the placebo (6 g/day olive oil), supplementation with the higher dose of EPA (1800 mg) but not DHA or a lower dose of EPA (600 mg) over 6 weeks can reduce Lp-PLA_2_ mass in a healthy population [39]. Apart from the effects in healthy subjects, an increase in n-3 intake has been shown to decrease Lp-PLA_2_ levels in patients with cardiovascular risk factors. In stable CAD patients undergoing percutaneous coronary intervention, the administration of omega-3 PUFA (1 g/day) for 4 weeks decreased Lp-PLA_2_ mass and activity compared to the control (soybean oil capsules) [40]. Similar findings have been observed in patients with diabetes [41] and subjects who had residual hypertriglyceridemia after receiving statins [42, 43].

The pro-atherogenic role of Lp-PLA_2_ could be related to its ability to hydrolyze oxidized phospholipids on the LDL-C surface, resulting in the generation of two proinflammatory and pro-apoptotic lipid mediators, lysophosphatidylcholine, and oxidized free fatty acids, which play an important role in the development of atherosclerotic necrotic cores by recruiting and activating macrophages or leukocytes [44]. In the present study, although canola oil reduced Lp-PLA_2_ levels, it had no significant effect on inflammatory factors. Consistent with this result, supplementation with n-3 fatty acids (EPA) reduced plasma Lp-PLA_2_ without having a significant effect on plasma inflammatory biomarkers, including IL-6 [39].

Complement factors C3 and C4 have been associated with atherosclerosis and cardiovascular risk factors [18] and have shown substantial correlations with cardiovascular risk factors [45]. Plasma C3 and C4 did not change in either group. In a previous study, an increase was observed in C3 concentration after the intake of high saturated fat compared with high-monounsaturated fat [14].

In the current study, neither olive oil nor canola oil consumption had a significant impact on plasma lipids or lipoproteins. Consistent with these findings, previous studies have reported no significant effect of the intake of refined OO (60 g/day) on plasma TG, LDL-C, or HDL-C levels in mildly hypercholesterolemic subjects who were not on lipid-lowering medications [46] or in stable coronary artery disease patients (50 mL/day) [47]. However, some previous studies have reported the beneficial effects of CO on plasma lipids [48–51]. Nevertheless, it should be noted that the study population in these studies were healthy people or those with cardiovascular risk factors who were not taking lipid-lowering agents. In contrast, most of the participants in the present study were under statin therapy and had an optimum level of plasma lipids. Therefore, the beneficial effects of canola oil on plasma lipid profiles appear to be evident in patients whose plasma lipids are high at baseline and are not on lipid-lowering medication. CO or OO intake in the context of a lipid-lowering diet for 3.5 weeks in hyperlipidemic subjects who were not on lipid-lowering agents had similar effects on serum lipoprotein concentration [22].

### Study strengths and limitations

The comparison of two commonly consumed edible oils and the detailed data collection were strengths of the current study; however, the present study also had some limitations. The study had no run-in period. Since participants received dietary advice on a heart-healthy diet at baseline, effects due to changes in dietary habits could not be excluded, and part of the observed effects on investigated biomarkers may be related to dietary habits modification. Participants of both groups received similar dietary advice, however, and the potential effects were present in both groups. The duration of dietary intervention was relatively short. Furthermore, some participants were lost to follow-up and/or discontinued the intervention, which reduced the final sample size. In addition, we were unable to blind the participants because of the sensory and appearance characteristics of the two oils. Moreover, the study could not be conducted in a tightly regulated, controlled feeding design. The study oils were provided to individuals (more than each participant’s need), and their consumption was monitored weekly. Because most participants were male, the sample cannot be considered representative, and this may limit the findings to a specific sex. Furthermore, the cross-over study design could have reduced the confounding factors associated with the inherited characteristics of the participants. Nevertheless, cross-over studies require that study participants be followed for a more extended period and that their treatment plan should not change during this time. Moreover, given that most of the population spends most of their time in the postprandial state, measurements of postprandial lipids and inflammatory markers could have provided additional information on potential changes in cardiovascular risk.

## Conclusions

In summary, each of the refined olive or canola oils improved one of the inflammatory CVD risk factors. Comparing the two vegetable oils in subjects with cardiovascular risk factors showed that the consumption of olive oil is more effective in reducing the level of inflammatory cytokine IL-6, whereas canola oil is more effective in lowering the level of LP-PLA_2_. Regarding the role of the circulating level of IL-6 in predicting future coronary heart disease events [52–54], a decrease in IL-6 level following olive oil consumption can reduce the risk of cardiovascular events. On the other hand, canola oil consumption decreased the level of Lp-PLA_2_, the increased levels of which have been associated with increased risk of cardiovascular events [16]. This finding should be interpreted with caution, however, because the Lp-PLA_2_ activity did not change significantly. Overall, the consumption of either refined olive or canola oils in the context of a healthy diet may have a beneficial effect on the secondary prevention of CVD by improving inflammatory risk factors.

## Data Availability

All data generated or analyzed during this study are included in this published article [and its supplementary information files].

## Abbreviations

ALA: Alfa-linolenic acid
ANCOVA: Analysis of covariance
CABG: Coronary artery bypass graft
CAD: Coronary artery disease
CO: Canola oil
CVD: Cardiovascular disease
DHA: Docosahexaenoic acid
EPA: Eicosapentaenoic acid
HDL-C: High-density lipoprotein cholesterol
IL-6: Interleukin-6
LDL-C: Low-density lipoprotein cholesterol
Lp-PLA_2_: Lipoprotein-associated phospholipase A_2_
MUFA: Monounsaturated fatty acid
OO: Olive oil
PUFA: Polyunsaturated fatty acid
SFA: Saturated fatty acid
TG: Triglycerides
